# Reduced T Cell and Antibody Responses to Inactivated Coronavirus Vaccine Among Individuals Above 55 Years Old

**DOI:** 10.3389/fimmu.2022.812126

**Published:** 2022-03-01

**Authors:** Giuliana X. Medeiros, Greyce Luri Sasahara, Jhosiene Y. Magawa, João Paulo S. Nunes, Fernanda R. Bruno, Andreia C. Kuramoto, Rafael R. Almeida, Marcelo A. Ferreira, Guilherme P. Scagion, Érika D. Candido, Fabyano B. Leal, Danielle B. L. Oliveira, Edison L. Durigon, Roberto Carlos V. Silva, Daniela S. Rosa, Silvia B. Boscardin, Verônica Coelho, Jorge Kalil, Keity S. Santos, Edecio Cunha-Neto

**Affiliations:** ^1^ Faculdade de Medicina da Universidade de São Paulo, Departamento de Clínica Médica, Disciplina de Alergia e Imunologia Clínica, São Paulo, Brazil; ^2^ Laboratório de Imunologia, Instituto do Coração (InCor), Hospital das Clínicas da Faculdade de Medicina da Universidade de São Paulo (HCFMUSP), São Paulo, Brazil; ^3^ Laboratório de Biologia Celular, LIM59, Departamento de Patologia da Faculdade de Medicina, Universidade de São Paulo, São Paulo, Brazil; ^4^ Departamento de Microbiologia, Instituto de Ciências Biomédicas, Universidade de São Paulo, São Paulo, Brazil; ^5^ Instituto Israelita de Ensino e Pesquisa Albert Einstein, Hospital Israelita Albert Einstein, São Paulo, Brazil; ^6^ Laboratório de Virologia, Plataforma Científica Pasteur da Universidade de São Paulo, São Paulo, Brazil; ^7^ Departamento de Microbiologia, Imunologia e Parasitologia, Universidade Federal de São Paulo (UNIFESP-EPM), São Paulo, Brazil; ^8^ Departamento de Parasitologia, Instituto de Ciências Biomédicas, Universidade de São Paulo, São Paulo, Brazil; ^9^ Instituto de Investigação em Imunologia (iii), Instituto Nacional de Ciências e Tecnologia (INCT), São Paulo, Brazil

**Keywords:** COVID-19, vaccine, CoronaVac, T cell responses, antibody, neutralizing antibody, age

## Abstract

CoronaVac is an inactivated SARS-CoV-2 vaccine that has been rolled out in several low and middle-income countries including Brazil, where it was the mainstay of the first wave of immunization of healthcare workers and the elderly population. We aimed to assess the T cell and antibody responses of vaccinated individuals as compared to convalescent patients. We detected IgG against SARS-CoV-2 antigens, neutralizing antibodies against the reference Wuhan SARS-CoV-2 strain and used SARS-CoV-2 peptides to detect IFN-g and IL-2 specific T cell responses in a group of CoronaVac vaccinated individuals (N = 101) and convalescent (N = 72) individuals. The frequency among vaccinated individuals, of whom 96% displayed T cell and/or antibody responses to SARS-CoV-2, is comparable to 98.5% responses of convalescent individuals. We observed that among vaccinated individuals, men and individuals 55 years or older developed significantly lower anti-RBD, anti-NP and neutralization titers against the Wuhan strain and antigen-induced IL-2 production by T cells. Neutralizing antibody responses for Gamma variant were even lower than for the Wuhan strain. Even though some studies indicated CoronaVac helped reduce mortality among elderly people, considering the appearance of novel variants of concern, CoronaVac vaccinated individuals above 55 years old are likely to benefit from a heterologous third dose/booster vaccine to increase immune response and likely protection.

## Introduction

Terminating the COVID-19 pandemic is dependent on global vaccination. CoronaVac (Sinovac, China) is a vaccine based on inactivated SARS-CoV-2 that has been deployed in China, Brazil, Indonesia, Thailand, Turkey, and Chile among other countries. It has been shown that CoronaVac’s immunogenicity is lower than natural infection (1). In Brazil, CoronaVac was the mainstay of the first wave of immunization of healthcare workers and the elderly population. Despite the finding of reduced COVID-19 mortality in Brazil among people above 70 or 75 years of age when CoronaVac was the most used vaccine, indicating protection for this group, immunogenicity of this vaccine in elderly individuals is still poorly known (2–4). Some studies reported seroconversion for up to 98% of vaccinated individuals, but anti-Spike antibody titers were significantly lower among those aged ≥60 years (5, 6). Also, the immunogenicity of inactivated vaccines such as Influenza have already been shown to be more limited among the elderly (7).

mRNA-based vaccines that protect more than 90% of the vaccinated individuals from severe COVID-19 were shown to induce T cell response (8, 9). Although an immunogenicity study in Chile has evaluated cellular immunity to CoronaVac, few patients were above 60 years of age (10). In order to assess the effect of age and sex in the vaccine response of adults and elderly people, we studied the anti-SARS-CoV-2 responses of a group of 101 vaccinated individuals (namely, 42 patients above 60). In this paper, we assessed T cell immune responses with an antigen-induced cytokine release assay (CRA) on whole blood and both binding antibody responses (against Spike, RBD and NP) and neutralizing antibodies against the original Wuhan strain.

## Materials and Methods

### Study Design and Participants

A cross-sectional study was performed with CoronaVac vaccinated health care workers, who reported no previous infection with SARS-CoV-2 (n = 101; median age = 55 IQR = 39–67); these subjects received two doses of 3 µg vaccine/shot, 3 weeks apart. The study was conducted at the Instituto do Coração in São Paulo, Brazil. Venous blood was collected at least 21 days (median = 37, IQR = 22–62) after the second immunization (**Table 1**). Convalescent individuals (confirmed by a previous positive SARS-CoV-2 RT-PCR result) with mild disease (11) (n = 72; median age = 40, IQR = 32–47) with at least 150 days since the onset of the infectious episode were included as positive controls. Seronegative samples with no T cell response specific for SARS-CoV-2, obtained during the pandemic (n = 36; median age = 36 IQR = 30–47), were also included as negative controls. All volunteers signed written informed consent and the study was approved by the Ethics Committee of the Hospital das Clínicas da Universidade de São Paulo (CAPPesq CAAE30155220.3.0000.0068).

**Table 1:** Characteristics of study participants.

	Vaccinated	Convalescent	Seronegative control	P value	
**n**	101	72	36	Test	
				A	p=4.37 x 10^-10^
				B	C vs SC p=0.94
**Age, mean ± sd**	54 ± 16.3	40.5 ± 10.5	39 ± 12.3		C vs V p<0.0001
**(range)**	(23-90)	(24-68)	(22-44)		SC vs V p<0.0001
**Sex, n (%)**					
** Female**	70 (71)	54 (75)	26 (72)	E	p=0.7129
** Male**	30 (29)	18 (25)	10 (28)		
**Interval between sampling and 2^nd^ dose or symptoms onset, median (range)**	37 (21-80)	207 (159-240)	NA		
**Antibody levels, median ratio (IQR)**					
** IgG RBD**	2.94 (3.04)	1.46 (1.00)	0.13 (0.09)	C	p=1.85 x 10^-20^
				D	C vs SC p<0.0001
					C vs V p=0.000147
					SC vs V p<0.0001
** IgG NP**	1.17 (2.69)	1.31 (1.34)	0.12 (0.14)	C	p=2.97 x 10^-15^
				D	C vs SC p<0.0001
					C vs V p=0.682
					SC vs V p<0.0001
** IgG Spike**	1.43 (3.03)	3.76 (2.28)	0.12 (0.11)	C	p=6.45 x 10^-18^
				D	C vs SC p<0.0001
					C vs V p<0.0001
					SC vs V p<0.0001
**Cytokine levels (pg/mL), median (IQR)**					
** IFN-γ**	2.06 (9.00)	3.51 (8.48)	0 (0.37)	C	p=1.18 x 10^-7^
				D	C vs SC p<0.0001
					C vs V p=1
					SC vs V p<0.0001
** IL-2**	1.21 (16.8)	2.72 (22.0)	0 (0.00)	C	p =2.7 x 10^-9^
				D	C vs SC p<0.0001
					C vs V p=0.326
					SC vs V p<0.0001
**Neutralization assay**					
**n**	83	31	36		
**Neutralization titer (VNT100), **	1:40	0.0972222	0		
**GMT (CI 95%)**	22.2 (15.2 - 32.4)	82.0 (56.7 - 118.6)	0	F	p = 0.0097

Tests: A, ANOVA; B, Post-hoc Tukey HSD; C, Kruskal-Wallis; D, Post-hoc Dunn; E, Chi square test.

F, Wilcoxon-Mann-Whitney.

Comparisons: V, vaccinated; C, convalescent; SC, seronegative control.

NA, Not applicable.

### Antibody ELISA

Enzyme-linked immunosorbent assay (ELISA) was performed using 96-well high- binding half-area polystyrene plates coated overnight at 4°C with 4 μg/ml of Spike protein, 2 μg/ml Nucleocapsid protein (NP) (Kindly provided by Dr. Ricardo Gazzinelli, UFMG, Brazil) or 0.8 μg/ml of the RBD domain from SARS-CoV-2 were all expressed in HEK293T cells (12). In short, 50 µl of diluted sera (1:100) were incubated at 37°C for 45 min. Peroxidase-conjugated goat anti-human IgG (BD Pharmingen,USA), anti-human IgA (KPL, USA) or anti-human IgM (Sigma, USA) secondary antibody conjugates were diluted 1:10,000, and incubated at 37°C for 30 min. The optical density (OD) at 492 nm was measured with a microplate reader (Epoch, BioTek, USA). Values were determined as OD minus blank and cutoff was determined as the average OD of 12 samples pre-pandemics + 3× standard deviation. Results are given as the ratio of OD sample/cutoff. An antibody ratio of ≥1.2 was considered positive.

### Virus Neutralization Assay

SARS‐CoV‐2 (GenBank: MT MT350282) was used to conduct a cytopathic effect (CPE)‐based virus neutralization test (VNT) as previously described (13). The virus strain properties were previously described on Araujo et al. (14). We used 96‐well plates containing 5 × 10^4^ cells/ml of Vero cells (ATCC CCL‐81). A series of dilutions (1:20 to 1:2,560) was prepared for the assay. Serum dilutions were mixed at equal volumes with the virus (100 tissue culture infectious doses, 100% endpoint per well—VNT100) and pre-incubated for virus neutralization for 1 h at 37°C. The mixtures containing serum and virus were transferred onto the confluent cell monolayer and incubated at 5% CO_2_ for three days at 37°C, all the procedures were conducted in a Biosafety laboratory level three (BSL3). After 72 h, plates were analyzed by light microscopy. Gross CPE was observed on Vero cells, distinguishing the presence/absence of CPE‐VNT against the Wuhan reference strain or the Gamma variant, which was prevalent in Brazil by the time of the sampling. To determine neutralizing antibody titers, the highest serum dilution that was able to neutralize virus growth was considered. As positive control, a reference serum from an RT‐qPCR positive individual and a plaque reduction in the neutralization test >640 was used in each assay.

### Antigen-Induced T Cell Cytokine Release Assay

Antigen-induced T cell cytokine release assays (CRA) were performed by incubating 250 µl/well of heparinized peripheral blood onto round-bottom 96-well plates for 48 h at a humidified 37°C, 5% CO2 environment in the presence of 20 pooled CD4^+^ T cell epitopes (**Supplementary Table 1**). Twenty CD4^+^ T cell epitopes used as antigen-specific T cell stimulus were bioinformatically identified and synthesized by scanning the whole proteome in SARS-CoV-2 reference genome (RefSeq: NC_045512.2) using the promiscuous HLA-DR binding peptide approach (15). Plates were centrifuged, and plasma supernatant was harvested and stored at −80°C until use. IFN-γ and IL-2 levels in cell-free culture supernatants were evaluated by ELISA, according to manufacturer’s instructions (R&D Systems, Minneapolis, MN). The cutoff values for IFN and IL-2 were obtained by a Receiver Operator Curve analysis with diagnostics as reference groups and IFN or IL-2 values as predictors. Cytokine values were subtracted from the DMSO control. The quantification limit of the IFN-γ test was 1.17 pg/ml and 0.98 pg/ml for IL-2.

### Statistical Analysis

Statistical analyses were performed using GraphPad Prism version 9 and R platform for statistical analysis version 4.0.3. Continuous variables were analyzed using Shapiro–Wilk test to assess the normality of their distribution. Comparison of continuous variables was carried out using Kruskal–Wallis test with Dunn’s *post-hoc* test for several groups or Mann–Whitney test when only two groups were compared. Pearson’s chi squared test was used to assess categorical data association. Correlation was evaluated by the Spearman’s coefficient. The age limit value to characterize groups of immune response was established after a two-step process. A K-means cluster analysis based on five continuous variables (ratios of IgG from NP, RBD, Spike and levels of IFN-γ and IL-2) was used to identify two groups of different immune responses, according to the algorithm of Hartigan and Wong (16). The age threshold that could distinguish the two resulting clusters with the highest accuracy was obtained by a Receiver Operator Characteristic curve analysis with the clusters as reference and the age variable as predictor (**Supplementary Figure 1**). A p-value <0.05 was considered statistically significant for all analyses.

## Results

**Figure 1:**
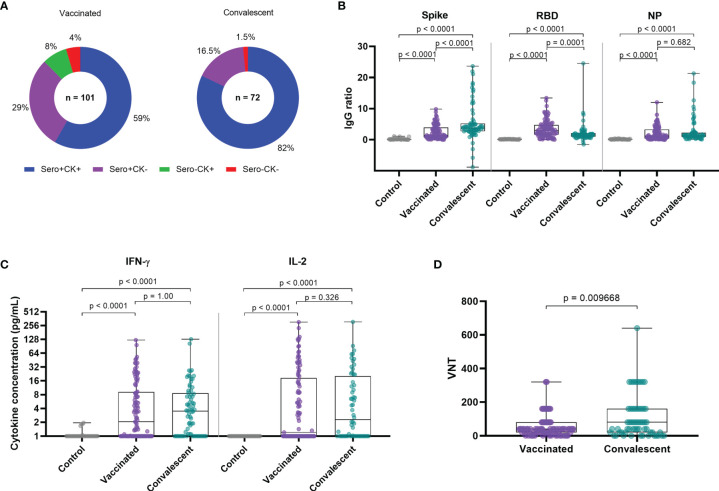
Immune responses among vaccinated and convalescent individuals. **(A)** IgG and T-cell SARS-CoV-2-specific cytokine production among vaccinated individuals and convalescents patients. **(B)** IgG reactivity against SARS-CoV-2 Spike protein, RBD domain and nucleocapsid protein. **(C)** T-cell SARS-CoV-2-specific cytokine release upon whole blood stimulation with specific SARS-COV-2 peptides. **(D)** Viral neutralization titers of original Wuhan strain. CK, Cytokine; VNT, Virus Neutralization Titer; NP, Nucleocapsid Protein from SARS-COV-2; IFN-g, Interferon gamma. Box plots show the median with IQR and the error bars indicate min and max values. VNT below 1:20 were considered 1 in graphs, numbers above the bars show the Geometric Mean Titer (GMT), and the error bars indicate the 95% CI. Statistical analysis: Kruskal–Wallis with Dunn *post hoc* test.

Demographical data and assays for each group are described in **Table 1**. Among vaccinated individuals, 96% displayed antigen-induced cellular cytokine and/or antibody responses to at least one antigen tested, and 98.5% of convalescents displayed cellular and/or antibody responses. Of interest, both cellular and humoral responses were displayed by 59 and 82% of vaccinated and convalescent individuals, respectively (**Figure 1A**). Vaccinated and convalescent individuals displayed significantly higher antibody and T cell responses than seronegative controls. Vaccinated individuals displayed significantly lower IgG responses against Spike protein, but higher responses against the RBD domain as compared to convalescent patients, while responses to NP were similar between those two groups (**Figure 1B**).

Regarding T cell responses as measured by cytokine release after peptide stimulation, we observed that IL-2 and IFN-γ levels were similarly increased among vaccinated individuals and convalescents in comparison to the seronegative control group (**Figure 1C**). Geometric mean titers (GMT) of neutralization titers for the vaccinated group were 4 times lower as compared to convalescent patients, even though time since infection was much longer than time after vaccination (**Figure 1D**). Of interest, time of sampling since vaccination or infection did not correlate with higher or lower immune responses (**Supplementary Figures 2**, **3**).

**Figure 2:**
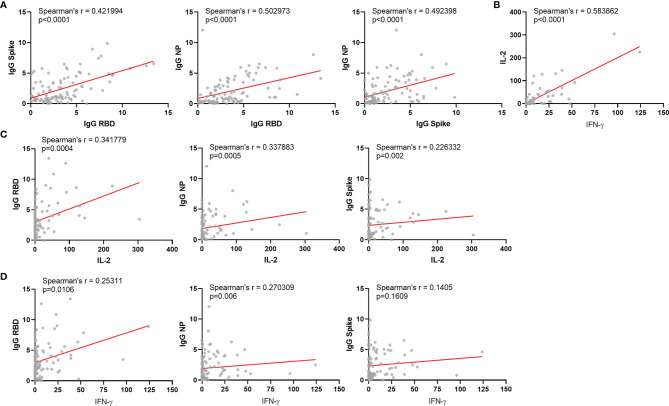
Correlations between different immunological parameters among vaccinated individuals. **(A)** Correlation between IgG for Spike protein and RBD; Correlation between IgG for NP protein and RBD; Correlation between IgG for NP protein and Spike protein. **(B)** Correlation between IL-2 and IFN-g released after whole blood stimulation. **(C)** Correlation between IgG for RBD, NP or Spike and IL-2 released after whole blood stimulation. **(D)** Correlation between IgG for RBD, NP or Spike and IFN- γ released after whole blood stimulation. NP: Nucleocapsid Protein from SARS-COV-2; IFN-γ: Interferon gamma. Spearman’s r and significances are indicated.

**Figure 3:**
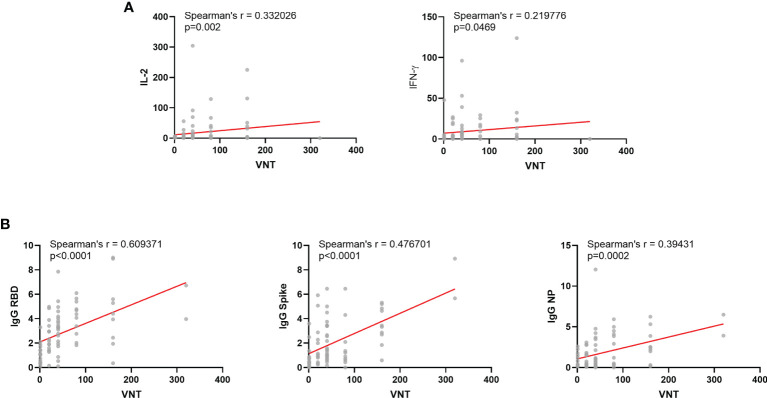
Correlations between Viral Neutralization Titers and other immunological parameters among vaccinated individuals. **(A)** Correlation between VNT and T-cell responses: IFN-γ and IL-2 released after whole blood stimulation. **(B)** Correlation between VNT and humoral responses: IgG for Spike, RBD or NP protein. VNT, Virus Neutralization Titer; NP, Nucleocapsid Protein from SARS-COV-2; IFN-g, Interferon gamma. VNT below 1:20 were considered 1 in graphs. Spearman’s r and significances are indicated.

**Figure 4:**
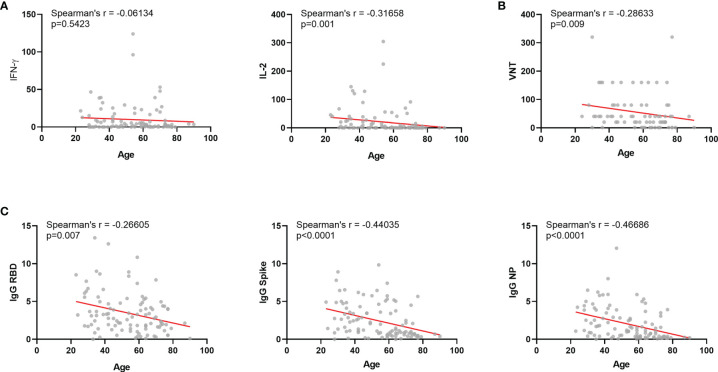
Correlations between age and other immunological parameters among vaccinated individuals. **(A)** Correlation between age and humoral responses: IgG for Spike, RBD or NP protein. **(B)** Correlation between age and T-cell responses: IFN-γ and IL-2 released after whole blood stimulation. **(C)** Correlation between age and VNT. NP, Nucleocapsid Protein from SARS-COV-2; IFN-γ, Interferon gamma; VNT, viral neutralization titers. Box plots show the median with IQR and the error bars indicate min and max values. Spearman’s r and significances are indicated.

Most immune response levels were positively correlated among each other, as previously reported (**Figure 2**) (17). Neutralization titers positively correlated with IgG levels for the three antigens tested and also with IFN-γ and IL-2 production upon whole blood stimulation (**Figure 3**). Among vaccinated individuals, we observed significant negative correlations between age and IL-2 cytokine release, but not for I IFN-γ (**Figure 4A**). We also observed a negative correlation between age and neutralization titers (**Figure 4B**), and also age and IgG antibody levels against Spike, RBD and NP (**Figure 4C**).

**Figure 5:**
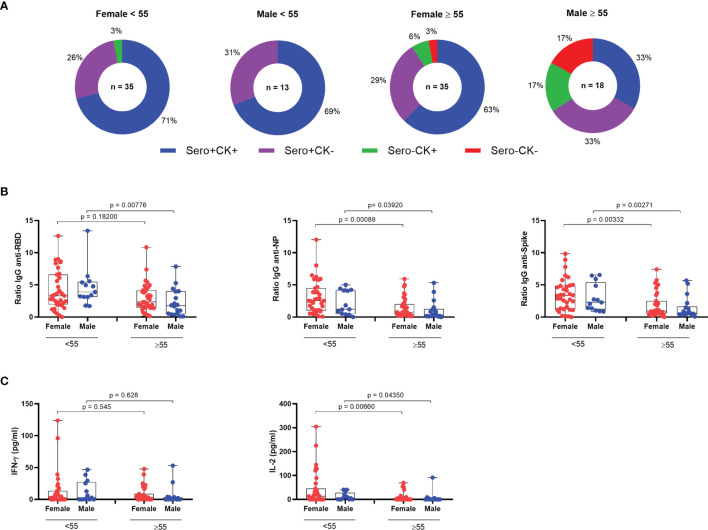
Immune responses among CoronaVac vaccinated individuals. **(A)** IgG and T-cell SARS-CoV-2-specific cytokine production among vaccinated individuals. (n = 101). **(B)** IgG reactivity against SARS-COV-2 Spike protein, RBD domain and nucleocapsid protein grouped by age and sex. **(C)** T-cell SARS-CoV-2-specific cytokine release upon whole blood stimulation with specific SARS-COV-2 peptides grouped by age and sex. NP, Nucleocapsid Protein from SARS-COV-2; IFN-γ, Interferon gamma. Box plots show the median with IQR and the error bars indicate min and max values. Statistical analysis: Wilcoxon rank sum test and Mann–Whitney test.

The identification of clusters from the numerical variables allowed us to identify the age of ~55 years as the best divisor. Therefore, we compared the two age groups considering under 55 and 55 or older. Among vaccinated, while 97% of the women ≥55 displayed antibody and/or T cell responses, 83% of men from the same age group displayed detectable responses (**Figure 5A**). Moreover, while 63% of women ≥55 displayed antibody and T cell responses simultaneously, only 33% of men in the same age group presented both types of response (**Figure 5A**). Antibody responses alone were observed in 33% vs 29% of men and women vaccinated individuals ≥55, respectively, and cellular responses in the absence of detectable IgG were found among 17% male and 6% female vaccinated individuals ≥55 years old (**Figure 5A**). Antibody responses for male vaccinated individuals ≥55 years old displayed the lowest levels of anti- Spike, anti-RBD and anti-NP IgG and IL-2 release (**Figures 5B, C**). Female vaccinated individuals ≥55 years old also showed lower anti-NP and anti-Spike IgG and IL-2 release than younger females (**Figures 5B, C**).

**Figure 6:**
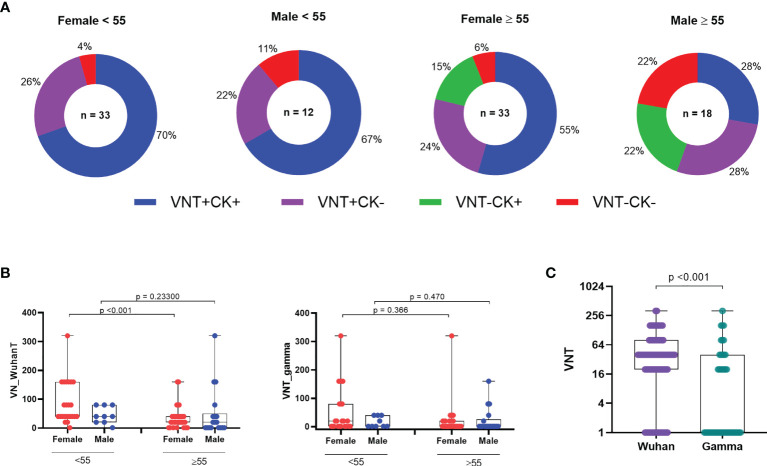
Frequency of immune responses of vaccinees considering VNT titers. **(A)** Distribution of VNT and/or CK responses among vaccinees. **(B)** Viral neutralization titers among vaccinated individuals grouped by age and sex (n = 83). **(C)** Viral neutralization of original Wuhan strain and VOC gamma. VNT, Virus Neutralization; VOC, variant of concern VNT below 1:20 were considered 1 in graphs, numbers above the bars show the Geometric Mean Titer (GMT), and the error bars indicate the 95% CI. Statistical analysis: Wilcoxon rank sum test and Mann–Whitney test.

Similar results were observed when we assessed neutralizing antibody and T cell cytokine production (**Figure 6A**). Older vaccinated men displaying both VNT and cytokine production corresponded to only 28% as compared to younger males presenting 67%, while among women, 70% of the younger and 55% of the older women presented both responses. Neutralization of Wuhan reference strain presented lower GMT for women >55 years old as compared with the younger group (**Figure 6B**). Lastly, we aimed to verify VNT capacity against the Gamma VOC, besides Wuhan strain, after vaccination. The overall VNT for the tested variant were lower than for Wuhan strain among vaccinated (**Figure 6C**). The dataset used for all analysis is available in **Supplementary Table 2**.

## Discussion

CoronaVac was the first anti-COVID vaccine available for mass immunization in Brazil, where it followed schedules targeting first the healthcare workers and the elderly population. We aimed to evaluate humoral and cellular responses after vaccination and stratify considering sex and age. In our study, the majority of vaccinated individuals developed some kind of immune response to the vaccine and the magnitude of most of the immune response measures positively correlated with each other. However, we observed a negative correlation between age and SARS-CoV-2-peptide epitopes antigen-induced IL-2 release and antibody responses to Spike, RBD and NP, suggesting a lower immunogenicity in older individuals. Upon stratification by age and sex, we observed that most of this reduced immunogenicity was among the ≥55 years male population. The proportion of individuals who displayed any response to the vaccine varied from 100% among younger women (<55 years old) to 83% among men 55 or older. Significantly, the simultaneous detection of antibody and cellular immune responses varied from 71% among women younger than 55 years old to only 33% among men 55 or older. This suggests that the immunogenicity of the vaccine regimen is less pronounced in this age and sex group. Also, the finding of reduced IL-2 in older male vaccinees is of particular concern, considering that interleukin-2 is essential for the development of memory T cells (18, 19).

Men are disproportionately affected by COVID-19 compared to their female counterparts. World death and hospitalization data during COVID-19 pandemic show a clear male bias (20). The mechanisms for such bias have not been elucidated, but it is fair to assume that differential immune responses play an important role. It has been hypothesized that this discrepancy is due to the fact that men generate a worse innate immune response than women. In regard to adaptive immunity, there is convincing evidence suggesting that women mount higher antibody responses (21), which is directly associated with a better vaccine response (22).

It is well reported that aging is associated with a decline in immune function [reviewed in (23)]. This process, known as immunosenescence, often leads to impaired response to vaccines in older adults. In the case of inactivated vaccines, such as influenza, elderly individuals have a significantly lower protection, with efficacy ranging from 17 to 51%, as compared to up to 90% for younger individuals (24).

COVID-19 immunogenicity across young and elderly individuals has been studied for other vaccines. For ChadOx1, no differences were found following the second dose either for anti-Spike IgG and neutralizing antibodies or for IFN-γ and IL-2 Th1 T cell responses among the 18–55, 55–69 and ≥70 year old groups (25, 26). A study between mRNA-1273 vaccine recipient groups of 56–70 or ≥71 years of age revealed that binding, neutralizing-antibody and IFN-γ and IL-2 responses were similar to those reported among vaccine recipients between the ages of 18 and 55 years and were above the median of a panel of controls who had donated convalescent serum (8, 27). As for the Pfizer BNT162b2 mRNA vaccine, one of the few studies comprising older adults (≥80 years old) showed a suboptimal neutralizing antibody response and reduced T cell count following the first dose (28). A second study reported that BNT162b2 elicited relatively lower antibody levels in adults over 50 years old vs younger adults (29). Lastly, responses among Chinese patients aged 18–55 and 65–85 showed similar IgG and somewhat lower neutralizing antibody and a more variable T cell response than younger individuals (30). T cell and antibody immune responses of the elderly groups after mRNA or adenovirus vector vaccines were thus found to be largely similar to those of younger individuals, which is in contrast with observations in our group of inactivated virus based CoronaVac vaccinated individuals. To our knowledge. this is the first report showing T-cell responses after vaccination with Coronavac with a significant group of older individuals. In our study, we used a whole blood IFN-γ and IL-2 release assay, while the cited studies used ELISPOT and flow cytometry analysis. Although ELISPOT could be considered more sensitive than the whole blood-based cytokine release assay used here, CRA has been shown to accurately assess cellular immunity to SARS-CoV-2 and vaccination (31–34). Of note, Tan et al. (35) performed a direct comparison between ELISPOT and CRA and found the sensitivity of the two methods are comparable with a strong positive correlation with each other. In addition, IgG antibody and VNT assays were similar to those used in the previous studies, and our results showed lower percentages among elderly individuals—especially in the male sex. This is especially relevant given the finding that CoronaVac vaccinated individuals were also shown to have significantly reduced neutralizing capacity against VOCs alpha, beta, and delta (1). The main VOCs circulating in Brazil at the time of sampling were gamma and alpha (36).

Our cluster analysis showed a difference in immune responses between older and younger of 55 years of age, which is not a common cutoff of age for comparison used in other studies. It shows that lower immune responses were detectable for people younger than 60 years and indicates that waning immunity could be just a matter of time after vaccination for these people. A nationwide evaluation of vaccine effectiveness comprising 25,752,013 Brazilians vaccinated with CoronaVac showed a reduced protection against hospitalization, ICU admission and death in individuals older than 79 years of age (67.2%), decreasing to 33.6% in individuals above 90 years ≥14 days after the 2nd dose. Sixty days after vaccination, there was an increase in the hospitalization rate in individuals >80 years old, indicating waning immunity and an eventual need for a booster dose for the elderly population (37).

Despite the important insight on the immunogenicity of CoronaVac, our study had limitations. The sample sizes were limited, and comorbidities were not considered for analysis in any group. Only 82% of participants had serum samples available for viral neutralization assay. Vaccinated individuals were included based on self-reporting no previous infection, but no basal antibody levels or SARS-CoV-2 RT-PCR were performed upon inclusion.

In summary, our results show a lower overall immune response for people older than 55 years after two-dose immunization with inactivated vaccine CoronaVac. In general, vaccinated subjects presented VNTs lower than convalescents for Wuhan strain and vaccination conferred lower VNTs against gamma VOC compared to Wuhan strain. Given the finding that mixing vaccines with different platforms may elicit stronger immunogenicity (38), our results corroborate the recommendation of the Brazilian Ministry of Health for a heterologous third dose/booster vaccine for elderly individuals vaccinated with CoronaVac.

## Data Availability Statement

The datasets presented in this study can be found in online repositories. The names of the repository/repositories and accession number(s) can be found in the article/**Supplementary Material**.

## Ethics Statement

The studies involving human participants were reviewed and approved by the Ethics Committee of the Hospital das Clínicas da Universidade de São Paulo (CAPPesq 79 CAAE30155220.3.0000.0068). The patients/participants provided their written informed consent to participate in this study.

## Author Contributions

Study design, data analysis, and critical review: SBB, VC, JK, KSS, and ECN. Data analysis and critical review: MAF. Data collection, data analysis, and critical review: GXM, GLS, JYM, FRB, JPSN, RRA, GPS, EDC, FBL, DBLO, ELD, RCVS, DSR. All authors listed have made a substantial, direct, and intellectual contribution to the work and approved it for publication.

## Funding

This paper has been funded by the Brazilian Ministry of Science and Technology, the Brazilian Science and Research Council (CNPq 465434/2014); the Sao Paulo State Science Foundation (Fapesp 2014/50890-5 and 2020/05256-7). The authors declare that this study received funding from JBS S.A. The funder was not involved in the study design, collection, analysis, interpretation of data, the writing of this article or the decision to submit it for publication.

## Conflict of Interest

The authors declare that the research was conducted in the absence of any commercial or financial relationships that could be construed as a potential conflict of interest.

## Publisher’s Note

All claims expressed in this article are solely those of the authors and do not necessarily represent those of their affiliated organizations, or those of the publisher, the editors and the reviewers. Any product that may be evaluated in this article, or claim that may be made by its manufacturer, is not guaranteed or endorsed by the publisher.

## Supplementary Material

Supplementary Figure 1 Receiver Operator Characteristic (ROC) curve used to establish the age threshold that could distinguish the two resulting clusters with the highest accuracy.

Supplementary Figure 2 Correlations between time post symptom onset and immunological parameters among convalescent individuals. **(A)** Correlation between time post symptom onset and T-cell responses: IFN-γ and IL-2 released after whole blood stimulation. **(B)** Correlation between time post symptom onset and humoral responses: IgG for Spike, RBD or NP protein.

Supplementary Figure 3 Correlations between time post 2^nd^ dose of CoronaVac and immunological parameters among vaccinated individuals. **(A)** Correlation between time post symptom onset and T-cell responses: IFN-γ and IL-2 released after whole blood stimulation. **(B)** Correlation between time post symptom onset and viral neutralization of original Wuhan strain. **(C)** Correlation between time post symptom onset and humoral responses: IgG for RBD, Spike or NP protein.
