# Supplementary figures and images for: Reduced T Cell and Antibody Responses to Inactivated Coronavirus Vaccine Among Individuals Above 55 Years Old
Source: Front Immunol. 2022 Mar 1;13:812126. doi: 10.3389/fimmu.2022.812126 (PMC8921991; doi:10.3389/fimmu.2022.812126)

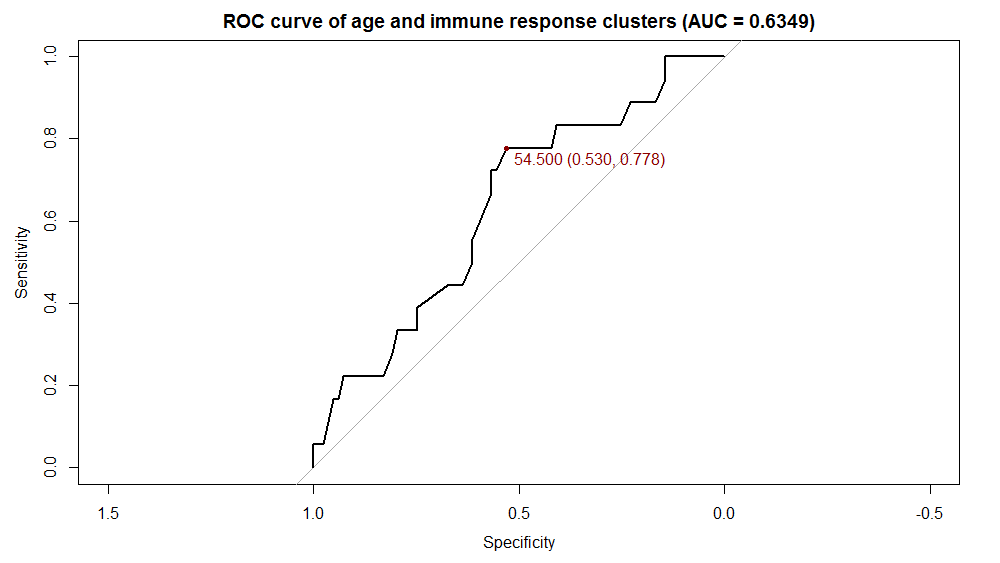

Supplement: Supplementary Figure 1 — Receiver Operator Characteristic (ROC) curve used to establish the age threshold that could distinguish the two resulting clusters with the highest accuracy. [file Image_1.tiff]

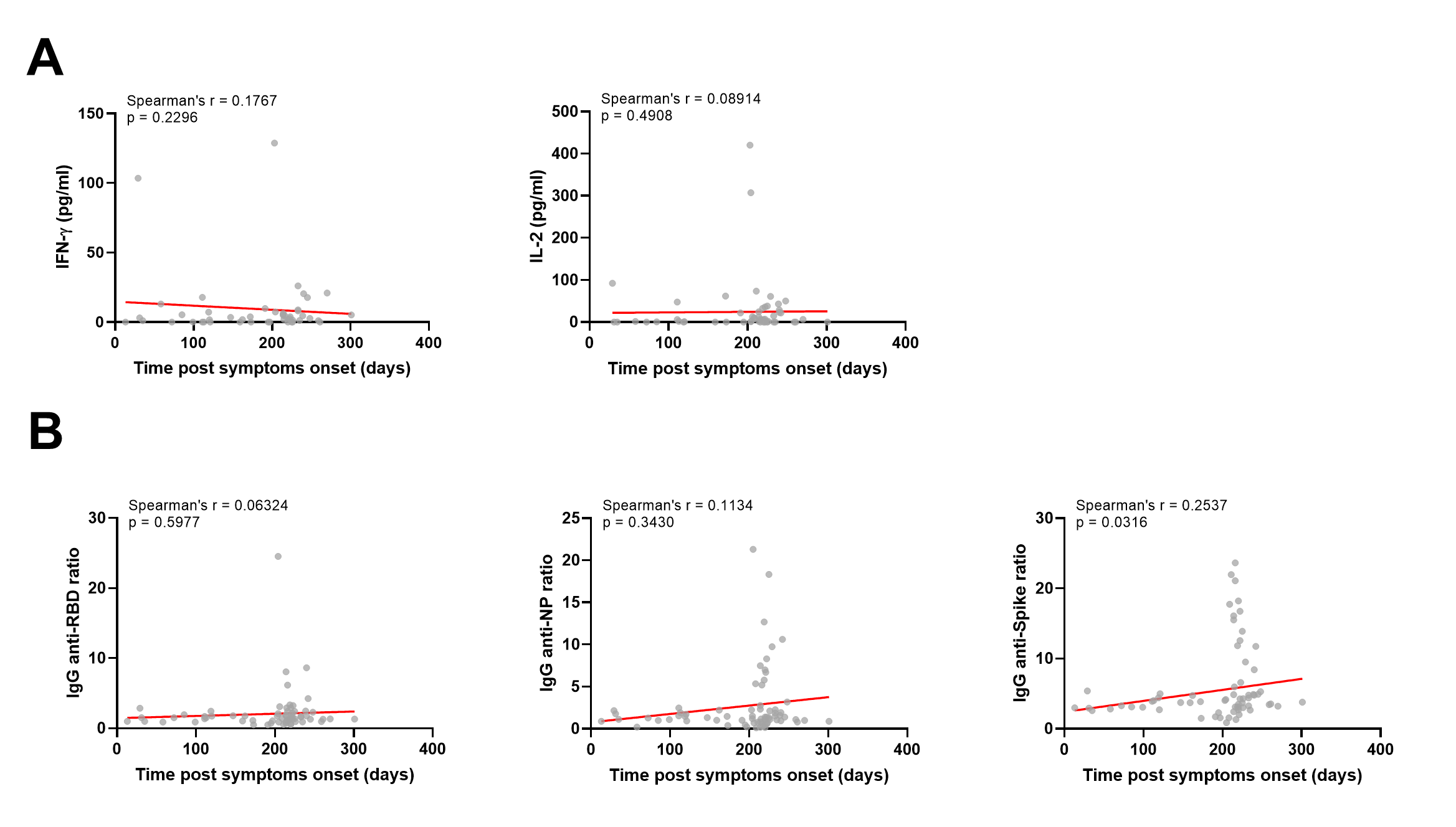

Supplement: Supplementary Figure 2 — Correlations between time post symptom onset and immunological parameters among convalescent individuals. (A) Correlation between time post symptom onset and T-cell responses: IFN-γ and IL-2 released after whole blood stimulation. (B) Correlation between time post symptom onset and humoral responses: IgG for Spike, RBD or NP protein. [file Image_2.tif]

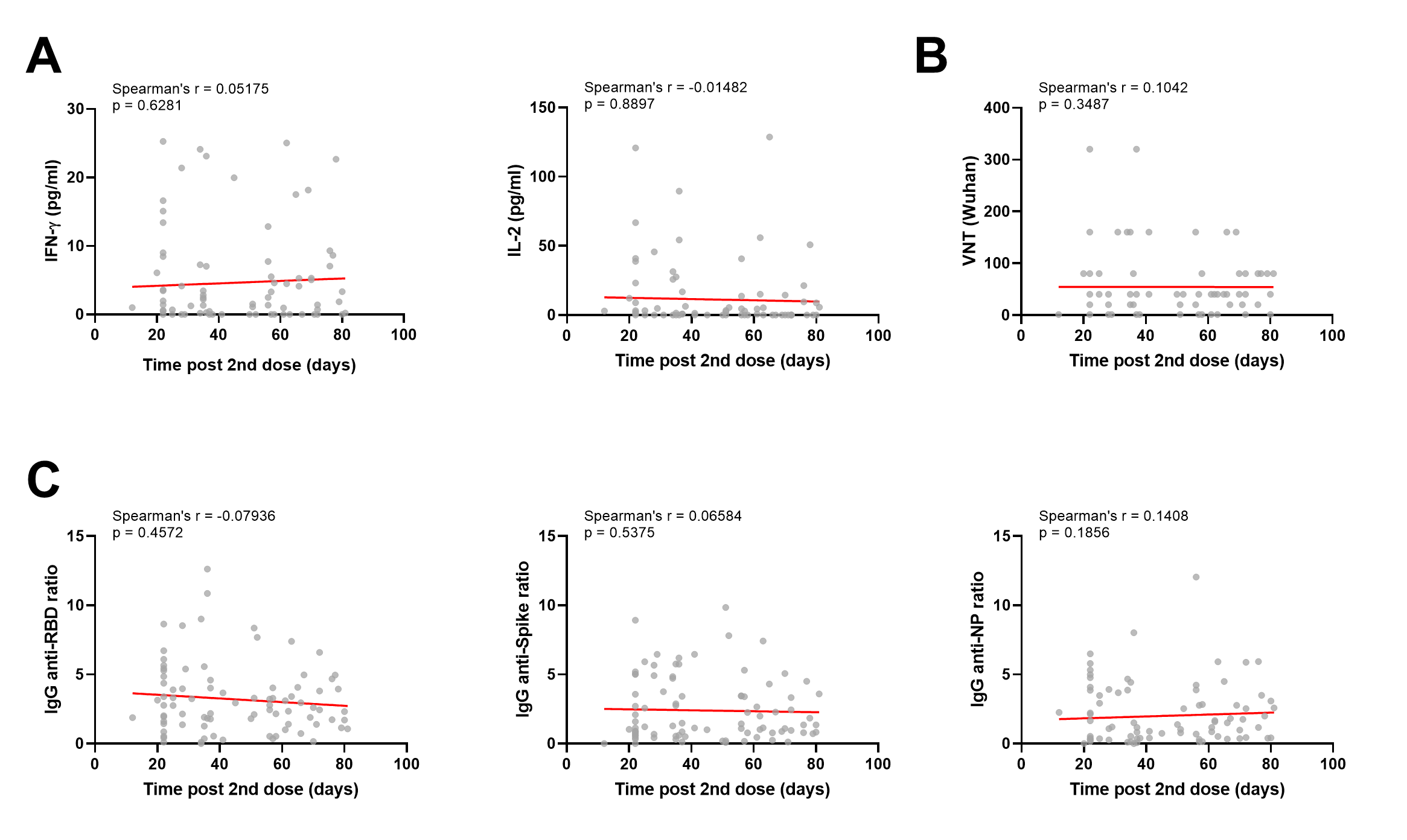

Supplement: Supplementary Figure 3 — Correlations between time post 2nd dose of CoronaVac and immunological parameters among vaccinated individuals. (A) Correlation between time post symptom onset and T-cell responses: IFN-γ and IL-2 released after whole blood stimulation. (B) Correlation between time post symptom onset and viral neutralization of original Wuhan strain. (C) Correlation between time post symptom onset and humoral responses: IgG for RBD, Spike or NP protein. [file Image_3.tif]
